# First Molecular Data of *Gongylonema pulchrum* (Rhabditida: Gongylonematidae) in European Fallow Deer *Dama dama* from Romania

**DOI:** 10.3390/pathogens13020175

**Published:** 2024-02-15

**Authors:** Dan-Cornel Popovici, Ana-Maria Marin, Ovidiu Ionescu, Maria Monica Florina Moraru, Durmuș Alpaslan Kaya, Mirela Imre, Narcisa Mederle

**Affiliations:** 1 Forestry Faculty, Transilvania University Brasov, 500123 Brasov, Romania; danpopovici30@yahoo.com (D.-C.P.); o.ionescu@unitbv.ro (O.I.); 2 Faculty of Veterinary Medicine, University of Life Sciences “King Michael I” from Timisoara, 300645 Timisoara, Romania; mariamoraru@usvt.ro (M.M.F.M.); mirela.imre@usvt.ro (M.I.); narcisamederle@usvt.ro (N.M.); 3 Faculty of Agriculture, Hatay Mustafa Kemal University, Hatay 31060, Turkey; dak1976@msn.com

**Keywords:** *G. pulchrum*, *D. dama*, Romania

## Abstract

Due to its adaptive versatility to numerous types of habitats, extremely diverse both in terms of composition and specificity, developed in various areas of the Western Plains of Romania, the European fallow deer (*Dama dama*) is a species with high ecological plasticity. In this area, the *D. dama* interacts with other species of wild fauna but also with numerous domestic animals, an important aspect in terms of the sanitary-veterinary status of animal populations, as well as the existence of a potential risk of infection with various species of parasites that can cause the *D. dama* specimens to obtain certain diseases and even zoonoses. A total of 133 esophagi from *D. dama* have been examined for helminths. Of the 133 esophagus samples from *D. dama*, nematodes of the genus *Gongylonema* were identified in 25 (18.80%). Sequencing revealed that the nematode identified in the samples was 99% similar to the sequence of *Gongylonema pulchrum* (GenBank no. LC026018.1, LC388754.1, AB646061). The present research is the first report of the nematode *G. pulchrum* from *D. dama* in Romania.

## 1. Introduction

The *D. dama* is a species of selenodonts (ruminants) within the Order Artiodactyla, of ungulates with equal toes, [1] totaling over 200 species globally, classified taxonomically in 10 distinct families. This species has been present in wild habitats in Romania since the Neolithic period and covers over 65% of Romania’s surface [2]. In Romania’s Western Plain, 60% of the *D. dama* herd is concentrated in Arad, Timiș, Bihor, Satu Mare, and Caraș Severin counties.

We appreciate that the *D. dama* is a species with high ecological plasticity and adaptive versatility to highly diverse habitats both in terms of composition and specificity, developed in various areas of the Western Plain. In this region, the *D. dama* comes into contact with various species of wild animals as well as domestic animals. These interactions are important in terms of the health of animal populations. There is a potential risk of infection with various parasites that can cause diseases to the *D. dama* specimens. Some of these diseases may also pose a risk to humans [3].

In Europe, infections with parasites belonging to the genera *Dicrocoelium*, *Paramphistomum*, *Fascioloides*, *Moniezia*, *Nematodirus*, *Oesophagostomum*, *Ostertagia*, *Toxocara*, *Trichostrongylus*, *Trichuris*, and *Eimeria*, as well as *Dictyocaulus* and *Protostrongylus* have been confirmed in *D. dama* [4,5,6,7,8]. In Romania, infections with gastrointestinal nematodes such as *Paramphistomum* spp., *Dicrocelium lanceatum*, and *Eimeria* spp. have been reported [9,10,11,12,13].

*Gongylonema* spp. is a widespread nematode throughout the world and affects domestic and wild mammals, birds, and occasionally humans. It is produced by species of the *Gongylonema* genus that are located in the mucosa of the upper digestive tract, including the tongue and especially the esophagus, producing white or red zigzag tracks in the mucosa [14]. *G. pulchrum* is a nematode with an evenly calibrated, long, and slender body. At the anterior extremity, it presents two cervical wings and four longitudinal rows of cuticular plates. The male is 3–6 cm × 0.3 mm, and the female is 8–14 cm × 0.5 mm [3].

The life cycle of the gullet worm is as follows: The intermediate hosts are the coprophagous cockroaches, in which the infective larval stage (L3) evolves in about 4 weeks. The definitive hosts acquire the infection via feeding on these insects or accidentally ingesting them with water and food [15]. Humans can become infected via accidentally ingesting the insect host or by drinking contaminated water [16,17].

In Romania, studies on *G. pulchrum* infection are poor. Popovici et al. [13] identified the presence of *Gongylonema* spp. eggs with a prevalence of 17% in the feces collected from *D. dama*. A prevalence of 42.8% of the nematode in roe deer has been reported [18]. Dărăbuș et al. [19] demonstrate the presence of the infection with the esophageal nematode in wild boar (*Sus scrofa)*. *S. scrofa* in the Republic of Moldova have a 5.8% prevalence of *G. pulchrum* infection, adding to the existing data [20]. There are no bibliographical references in Romania regarding the molecular identification of the species *G. pulchrum* in *D. dama*. The aim of this study was to investigate the occurrence of *G. pulchrum* in *D. dama* and to molecularly characterize the species, which is being reported for the first time in Romania.

## 2. Materials and Methods

This study was carried out between October 2021 and February 2023, on 133 *D. dama* (62 males and 71 females, respectively), from different hunting funds in eight counties of Romania. The animals were hunted in accordance with the annual harvest quotas set by the Ministry of Environment, Water, and Forests [21]. The establishment of these quotas was carried out on the basis of the hunting management criteria and was continued with the extraction of fallow deer specimens by sex, specimen quality, and age categories. Afterward, the esophagus was taken from each individual and examined for gongylonemosis in the Parasitic Diseases Clinic of the Faculty of Veterinary Medicine/University of Life Sciences “King Mihai I” in Timișoara, Romania.

### 2.1. Necropsy Examination

The esophagus from each *D. dama* under study was sectioned with laboratory scissors. After sectioning, for proper examination, each esophagus was examined under a stereomicroscope. Parasites were removed from the esophagus with eye forceps, counted, and stored in 96% ethanol for molecular analysis.

### 2.2. PCR Protocol

Samples consisting of adult nematodes were collected and subjected to molecular analysis for the identification of parasite DNA. This extraction was carried out using the BIoline Tissue Protocol Kit (BIOLINE^®^ UK Ltd., London, UK). The DNA was stored at −20 °C until further analysis.

#### Polymerase Chain Reaction (PCR)

The PCR reaction was performed according to the technique described by da Silva et al. [22] and Gasser et al. [23], with some minor modifications. The amplification was performed using classical PCR, targeting the ITS gene sequence of ~850 bp size. The primers used were: NC5 forward (5′-GTAGGTGAACCTGCGGAAGGATCATT-3) and NC2 reverse (5′ TTAGTTTCTTTTCCTCCGCT-3′).

Amplification was carried out according to the protocol described, modified according to the requirements of the mixture. Master Mix MyTaqTM Red Mix (BIOLINE^®^ UK Ltd., London, UK) was used to perform the reaction. The final volume of the PCR reaction was 25 µL, of which 12.5 µL was MyTaqTM Red Mix (BIOLINE^®^), 1 µL was NC5 primer, and 1 µL was NC2 primer (diluted to a concentration of 10 pmol/µL, according to the protocol described by the manufacturer), DNA (extracted from the sample) and ultrapure water.

The amplification program was carried out with the My Cycler thermocycler (BioRad^®^, Berkeley, CA, USA). This program included DNA denaturation at 95 °C for 1 min; 35 cycles of denaturation at 95 °C for 30 s; hybridization at 50 °C for 30 s; and extension at 72 °C for 30 s, followed by incubation at 4 °C.

The analysis and control of the amplicons was performed using horizontal electrophoresis in a submerged electrophoresis system in 1.5% agarose gel, with the addition of the fluorescent dye RedSafe™ (iNtRON Biotechnology, Inc., Gyeonggi-do, Korea), at a voltage of 120 V and 90 mA, for 60 min. The 100 bp DNA ladder marker (BIOLINE^®^ UK Ltd., London, UK) was used in the first well of the gel. After migrating the samples in the agarose gel, the image of the gel with the migrated DNA fragments was captured using a UV photo documentation system (UVP^®^).

To confirm the species, a total of three isolates from the PCR products were sequenced in the forward and reverse direction by the company Macrogen Europe B.V., Amsterdam, The Netherlands. A homology search was performed using the online version of the Basic Local Alignment Search Tool (BLAST) software (available at: https://blast.ncbi.nlm.nih.gov/Blast.cgi, accessed on 10 January 2024).

Phylogenetic analysis using the ITS gene sequences, isolated from samples in the present study, and those of the same genus retrieved from the GenBank databases were used. The accession number of the sequences analyzed in the present study is colored blue in the figure, showing the phylogenetic tree (Supplementary file S2). Multiple alignments of the nucleotide sequences of the haplotypes were performed using the Clustal W algorithm. Maximum likelihood (ML) analysis was performed with the program PhyML [24,25] provided on the ‘phylogeny.fr’ website (available at: http://www.phylogeny.fr/, accessed on 6 February 2024).

## 3. Results

**Figure 1:**
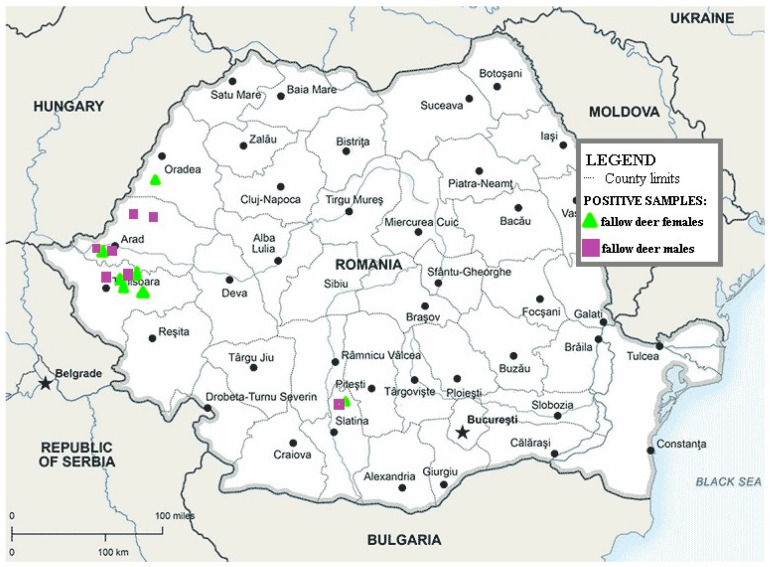
Map showing the geographical areas where *D. dama* were collected; green triangle shows the sites where female positive animals were found and purple squares shows the sites where male positive animals were found.

Out of the 133 esophageal samples, *Gongylonema* nematodes were identified in 18.80% of the *D. dama* samples from four Romanian counties (Olt, Timiș, Arad, and Bihor) (Figure 1).

**Figure 2:**
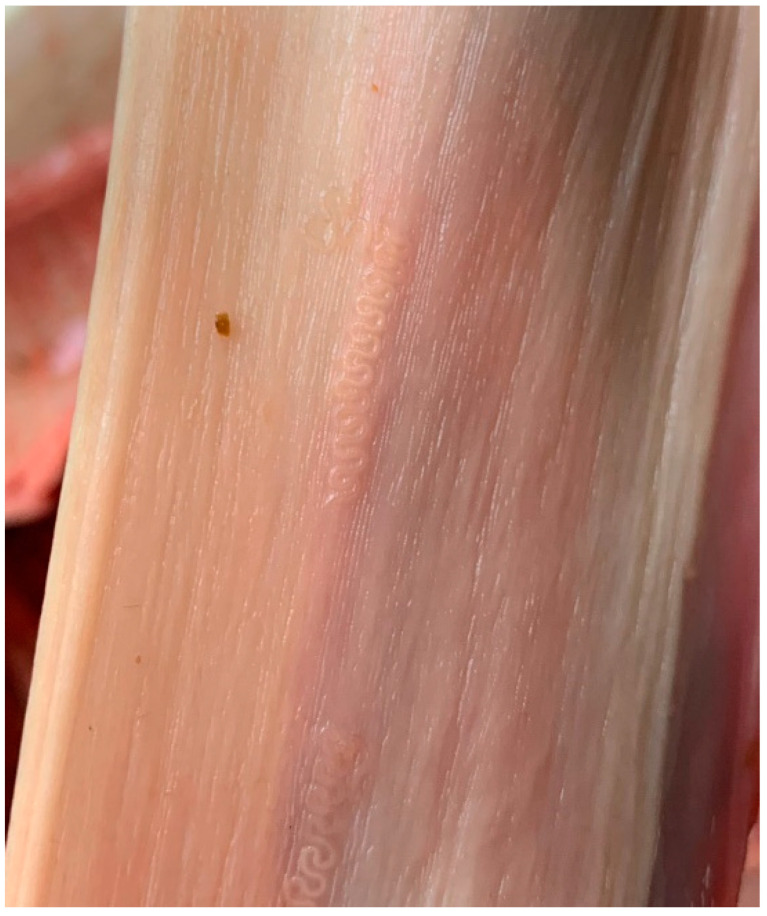
Esophagus of *D. dama* infected with *G. pulchrum*.

The parasitism with the gullet worm was diagnosed in the samples examined with a different prevalence depending on the season: 56% (14/25) in autumn, and 44% (11/25) in winter. Parasites were identified throughout the esophagus. Positive esophagus samples contained a mean intensity (25.4) and intensity range (2–73) parasites per sample. Based on the localization and morphological characteristics according to Soulsby [26], the parasites were identified as *G. pulchrum* (Figure 2). Species identification of parasites isolated from the esophagus of *D. dama* was performed using molecular biology. PCR amplification revealed clear bands at ~850 bp. The samples were cleaned using the commercial kit ISOLATE II PCR and Gel Kit (Bioline, London, UK) according to the manufacturer’s protocol and sent to be sequenced. Sequencing revealed that the nematode identified in the samples was 99% similar to the sequence of *G. pulchrum* (GenBank no. LC026018.1, LC388754.1, AB646061). The sequence was deposited in GenBank with accession number PP229209.1 (Supplementary File S1). The phylogenetic analysis in Supplementary File S2 reveals the homology with other *G. pulchrum* isolates like LC026018.1, LC388754.1, and AB646061 identified in wild or domestic ruminants.

## 4. Discussion

The ecological interaction between domestic and wild mammals and their susceptibility to different species of the genus *Gongylonema* must be taken into account when trying to elucidate the transmission dynamics of this nematode in nature [14,25]. The present study reports a prevalence of 18.80% (25/133) of gullet worm infection in 133 specimens of *D. dama*, collected from eight counties of Romania in the 2021/2022 and 2022/2023 hunting seasons.

Infections with esophageal nematodes and gastrointestinal nematodes (*Capillaria bovis*, *Cooperia punctata*, *Haemonchus contortus*, *Trichostrongylus axei*, *Ostertagia spp.*, and *Oe. venulosum*) have been identified in both domestic and wild ruminants, but also in *S. scrofa* in Hawaii and Spain [27,28], Texas [29], Pakistan [4], Iran [30], Turkey [31], and Japan [32]. In Romania, studies related to *D. dama* parasite fauna were carried out by Darabus et al., 2009 [9], Hora et al., 2017 [10], and Popovici et al. [11,12,13].

Infection with *G. pulchrum* associated with severe acanthosis is diagnosed in 4.57% and 7.6% of sheep (*Ovis aries*) examined in Iran [33,34]. The limits of the prevalence of *Gongylonema* spp. in cattle (*Bos domesticus*), in Iran, oscillate between 0.8% [35] and 16.2% [15]. *B. domesticus* from the Dagestan region (Russia) showed a prevalence of *G. pulchrum* infection of 45.22% [36], and in goats (*Capra aegagrus hircus*) from the Sanliurfa region (Turkey), the nematode reached a prevalence of 32.53% [37].

Wild ruminants also have bibliographic sources from America and Europe. Thus, the species *G. pulchrum* (57.9%) and *G. verrucosum* (16.6%), as well as infection with *Paramphistomum liorchis* (7.3%) were identified in roe deer (*Capreolus capreolus*) from south-eastern USA [38]. In Bulgaria, *G. pulchrum* infection in *D. dama* was reported for the first time by Yanchev in 1979, and later by Todev et al. in 2004, and Nanev et al. in 2010 [8]. Esophageal infection was also diagnosed in other hosts: Tibetan macaques (*Macaca thibetana*) (prevalence of 31.58%) [39], rats (*Rattus norvegicus*) (20%) [40], donkey (*Equus africanus asinus*) [41], the wild rabbit (*O. cuniculus*) [42], and brown-nosed coati (*Nasua nasua*) from Brazil [43].

The results regarding the number of parasites quantified in each of the 25 positive esophagus samples from *D. dama* in the present study revealed an average of 25.4 nematodes with limits between 2 and 73 *Gongylonema* spp. Compared to other host animals, Eslami et al. conducted a study in which they examined 350 *O. aries* esophagus samples, of which 16 were positive for *G. pulchrum* infection and harbored between 1 and 100 worms with an average of 10 [33]. Eira et al. [42] examined 112 *O. cuniculus*, of which 14 were positive for *G. pulchrum* infection and harbored between 1 and 10 worms with a mean of 2.86. The structure of the results of the phylogenetic tree highlighted that our isolate was grouped in a distinct clade, together with other representative GenBank-deposited *G. pulchrum* sequences, isolated from various hosts in different countries and from several geographical regions of the world. Evolutionary distance analysis indicates an affined bootstrap support level between the isolates and discrimination from other different species of the infraorder Spiruromorpha.

The study confirms the presence of *G. pulchrum* in Romania for the first time using molecular biology. The species was isolated from the esophagus of a *D. dama*. *G. pulchrum* and *G. nepalensis*, a newly identified species, are widespread in various domestic and wild mammals from Japan, Nepal, Sardinia, and Italy [25,32,44,45,46]. Rodents in Tunisia and Southeast Asia, and *O. cuniculus* in Portugal are definitive hosts for the potentially zoonotic species *G. neoplasticum* [42,47].

The first recorded infection of *G. scutatum* in domestic ruminants was mentioned in 1915 by Ransom and Hall [48]. A recent study conducted by Varcasia et al. in 2017 [46] has indicated for the first time that the *G. nepalensis* species is present in domestic ruminants and mouflon (*Ovis ammon musimon*). The morphological and molecular characterization supports *G. pulchrum* infection in 0.53% of the *B. domesticus* examined in Turkey between November 2017 and June 2019 [49]. Adult owls and their young appear as accidental hosts for *Gongylonema* spp., the nematode that causes necrotic oropharyngeal disease in these hosts [50,51,52].

Sporadic zoonotic infections have been diagnosed on all continents [16,17]. In total, 50 cases were reported worldwide in 2012, of which 11 were reported in the USA [53], the first case, in France, in 2013 [54], and in Slovenia in 2019 [55]. In 2021, the first case of comorbidity, early esophageal cancer and esophageal gongylonemosis, was reported in a patient in whom an unknown connection between the parasitic infection and the tumor condition was speculated [56]. In 2024, in China, a new case of *G. pulchrum* infection was reported in a patient who raised sheep, thus underlining the authors’ assertions that support the increased risk of contamination in people who live in the vicinity of sheep and cattle farms [16]. A novel, intraocular localization is described by Waisberg et al. [57], in 2018 in Brazil, in a patient who used to consume unfiltered water.

The presence of intermediate hosts associated with the size of the host population, as well as the characteristics of the microclimate, are important risk factors in the parasitic process and evolution [58,59]. The intermediate hosts involved in the transmission of infection with the *Gongylonema* spp. are dung beetles (family Scarabeidae), which, through their biological behavior, play an important role in the life cycle of other helminths [60]. Eight species belonging to the Scarabeidae family were identified as intermediate hosts for *G. pulchrum* both on the pastures where domestic animals live, as well as on three pastures occupied by fallow deer and mouflons [61].

In the present study, the infection with the gullet worm that affected the 25 specimens of *D. dama* from four counties of Romania (Olt, Timis, Arad, and Bihor) was diagnosed in the samples examined in autumn 56% (14/25) and in winter 44% (11/25). Comparatively, a study conducted on *O. aries* indicates a higher prevalence in summer (10.4%) and lower in autumn (6.2%) [34]. In Iran, some authors claim that *Gongylonema* infection affects *B. domesticus* with a high prevalence in summer and a low prevalence in winter [15], but there are also authors who advocate for the opposite situation [62]. Accordingly, in primates (macaques), Yang et al. [63] indicate a higher prevalence in summer (86.21%), compared to the cold season (7.14%).

The wide plethora of definitive hosts susceptible to infection with *G. pulchrum* (mammals, birds, and humans), the possibilities of interference between the microclimate of wild ruminants, and the pastures occupied by domestic animals through the circulation of coprophagous insects (intermediate hosts common to the sylvatic and domestic environment), but also of rodents (definitive hosts), indicates the existence of an epidemiological context favorable to the development of parasitic elements and a warning for domestic animal breeders and, implicitly, for humans.

## 5. Conclusions

The prevalence of esophageal infection in 133 *D. dama* specimens collected from eight counties in Romania between 2021–2022 and 2022–2023 hunting seasons was found to be 18.80%. The isolated nematode species from the *D. dama* esophagus was identified as *G. pulchrum* through molecular characterization. It is the first report in Romania of the molecular identification of the species *G. pulchrum* in *D. dama*. The study opens up the possibility of future research and warns specialists, animal breeders, and hunters about the risk of infestation with the esophageal nematode that affects mammals, birds, and humans.

## Data Availability

All data related to this study are presented and published here.

## Supplementary Materials

The following supporting information can be downloaded at: https://www.mdpi.com/article/10.3390/pathogens13020175/s1, File S1: Sequence deposited in GenBank with accession number PP229209.1; File S2: The phylogenetic analysis of *Gongylonema* isolates deposited in GenBank.
