# Supplementary material for: First Molecular Data of Gongylonema pulchrum (Rhabditida: Gongylonematidae) in European Fallow Deer Dama dama from Romania
Source: Pathogens. 2024 Feb 15;13(2):175. doi: 10.3390/pathogens13020175 (PMC10892342; doi:10.3390/pathogens13020175)

Supplementary file S1

An official website of the United States government

Here's how you know

Nucleotide

GenBank

Gongylonema pulchrum isolate RO_465 internal transcribed spacer 1, partial sequence; 5.8S ribosomal RNA gene, complete sequence; and internal transcribed spacer 2, partial sequence

GenBank: PP229209.1

FASTA Graphics

Go to:

```
LOCUS       PP229209                 485 bp    DNA     linear   INV 03-FEB-2024
DEFINITION  Gongylonema pulchrum isolate RO_465 internal transcribed spacer 1,
            partial sequence; 5.8S ribosomal RNA gene, complete sequence; and
            internal transcribed spacer 2, partial sequence.
ACCESSION   PP229209
VERSION     PP229209.1
KEYWORDS    .
SOURCE      Gongylonema pulchrum
  ORGANISM  Gongylonema pulchrum
            Eukaryota; Metazoa; Ecdysozoa; Nematoda; Chromadorea; Rhabditida;
            Spirurina; Spiruromorpha; Spiruroidea; Gongylonematidae;
            Gongylonema.
REFERENCE   1  (bases 1 to 485)
  AUTHORS   Imre,M., Popovici,D.C., Marin,A.-M., Ionescu,O., Moraru,M.M.F.,
            Kaya,D.A. and Mederle,N.
  TITLE     Molecular Characterization of Gongylonema pulchrum (Molin 1857) in
            Fallow Deer in Romania - First Report
  JOURNAL   Unpublished
REFERENCE   2  (bases 1 to 485)
  AUTHORS   Imre,M., Popovici,D.C., Marin,A.-M., Ionescu,O., Moraru,M.M.F.,
            Kaya,D.A. and Mederle,N.
  TITLE     Direct Submission
  JOURNAL   Submitted (27-JAN-2024) Parasitology and Parasitic Disease
            Department, University of Life Sciences Timisoara, Timisoara, Calea
            Aradului No. 119, Timisoara, Timis 300645, Romania
COMMENT     ##Assembly-Data-START##
            Sequencing Technology :: Sanger dideoxy sequencing
            ##Assembly-Data-END##
FEATURES             Location/Qualifiers
     source          1..485
                     /organism="Gongylonema pulchrum"
                     /mol_type="genomic DNA"
                     /isolate="RO_465"
                     /isolation_source="esophagus"
                     /host="Dama dama"
                     /db_xref="taxon:637853"
                     /country="Romania"
                     /collection_date="01-Sep-2022"
     misc_RNA        <1..>485
                     /note="contains internal transcribed spacer 1, 5.8S
                     ribosomal RNA, and internal transcribed spacer 2"
ORIGIN
        1 cccccacaaa aaaaaaaaaa caccccccac acacacacac acattattgt attttggtaa
       61 aaatatactg ataaaaatat cagttataaa gagatttact cttagcggtg gatcacttgg
      121 ctcgtggatc gatgaagaac gcagctagct gcgataaata gtgcgaattg cagacacatt
      181 gagcactaaa atttcgaacg cacattgcgc catcgggttc attcccgttg gcatgtctgg
      241 ctgagggtta attcaaacaa aaagaatcta tctcagattg aatatgaaaa accatcgtca
      301 ggtaatgtga tggttgtact gctgctgcgg ctgctatatt tccctgatca gtttttgcta
      361 ttataatgtt gtacggggca acaacaattc aaatagcaac tattaatttc tactactaca
      421 acaaagaaag aaaatttaca tgtatgtagt agagaaaaga atgtggtgcg cattattaat
      481 tttta
//
```

Supplementary file S2

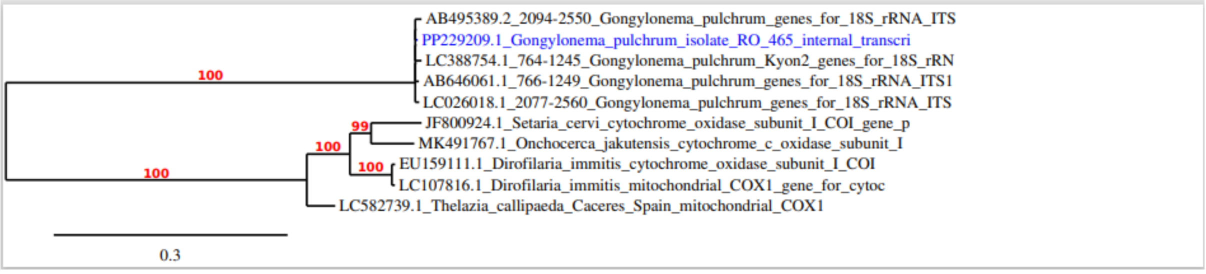

Supplement: Supplementary file 1 [file pathogens-13-00175-s001.zip › pathogens-2852361-supplementary.pdf]
